# Three-Dimensional Radiomics Features of Magnetic Resonance T2-Weighted Imaging Combined With Clinical Characteristics to Predict the Recurrence of Acute Pancreatitis

**DOI:** 10.3389/fmed.2022.777368

**Published:** 2022-03-10

**Authors:** Yuntao Hu, Nian Liu, Lingling Tang, Qianqian Liu, Ke Pan, Lixing Lei, Xiaohua Huang

**Affiliations:** Department of Radiology, Affiliated Hospital of North Sichuan Medical College, Nanchong, China

**Keywords:** radiomics, magnetic resonance imaging, T2-weighted imaging, recurrent, acute pancreatitis

## Abstract

**Objective:**

To explore the diagnostic value of radiomics model based on magnetic resonance T2-weighted imaging for predicting the recurrence of acute pancreatitis.

**Methods:**

We retrospectively collected 190 patients with acute pancreatitis (AP), including 122 patients with initial acute pancreatitis (IAP) and 68 patients with recurrent acute pancreatitis (RAP). At the same time, the clinical characteristics of the two groups were collected. They were randomly divided into training group and validation group in the ratio of 7:3. One hundred thirty-four cases in the training group, including 86 cases of IAP and 48 cases of RAP. There were 56 cases in the validation group, including 36 cases of IAP and 20 cases of RAP. Least absolute shrinkage and selection operator (LASSO) were used for feature screening. Logistic regression was used to establish the radiomics model, clinical model and combined model for predicting AP recurrence. The predictive ability of the three models was evaluated by the area under the curve (AUC). The recurrence risk in patients with AP was assessed using the nomogram.

**Results:**

The AUCs of radiomics model in training group and validation group were 0.804 and 0.788, respectively. The AUCs of the combined model in the training group and the validation group were 0.833 and 0.799, respectively. The AUCs of the clinical model in training group and validation group were 0.677 and 0.572, respectively. The sensitivities of the radiomics model, combined model, and clinical model were 0.646, 0.691, and 0.765, respectively. The specificities of the radiomics model, combined model, and clinical model were 0.791, 0.828, and 0.590, respectively. There was no significant difference in AUC between the radiomics model and the combined model for predicting RAP (*p* = 0.067). The AUCs of the radiomics model and combined model were greater than those of the clinical model (*p* = 0.008 and *p* = 0.007, respectively).

**Conclusions:**

Radiomics features based on magnetic resonance T2WI could be used as biomarkers to predict the recurrence of AP, and radiomics model and combined model can provide new directions for predicting recurrence of acute pancreatitis.

## Introduction

Recurrent acute pancreatitis (RAP) is an important global clinical problem (1). It is controversial whether RAP is an extension of acute pancreatitis (AP) or an aggravation of chronic disease (2). However, in recent years, most studies have shown that AP can be developed into RAP and eventually into chronic pancreatitis (CP) (3, 4). About 22% of initial AP patients (IAP) develop RAP. Ten percent of IAP patients and 36 percent of relapsing AP patients developed CP (5). Therefore, the prevention of the recurrence of acute pancreatitis is the key to preventing the development of acute pancreatitis into chronic pancreatitis (6, 7).

At present, the clinical etiology of recurrent acute pancreatitis has been gradually clarified and include choledocholithiasis or sludge and cholestasis, Oddi sphincter dysfunction, obstruction of the main pancreatic duct or bile duct junction, anatomic ductal variation interfering with pancreatic fluid outflow, genetic mutations, and excessive alcohol consumption. For example, a study (8) found that the pancreatic volume and tail diameter in patients with acute pancreatitis recurrence were smaller than those in normal subjects as assessed by axial T1-weighted imaging (T1WI) magnetic resonance imaging (MRI). Using magnetic resonance cholangiopancreatography, they (9) found that the meandering main pancreatic duct was more likely to cause RAP. Although CT and MRI can visually improve the diagnosis rate of AP, the etiology of up to 30% of RAP cases remains unknown (10). Therefore, it is difficult to predict recurrence of AP from a clinical perspective alone.

Radiomics provides a non-invasive method to capture a large amount of in-depth information about the heterogeneity of lesions that cannot be observed by the naked eye. Then, by combining imaging data from many patients with complex bioinformatics tools, models can be developed that significantly improve diagnosis and diagnostic accuracy (11). Another study (12) found that the radiomics model based on CT imaging also predicts the recurrence of acute pancreatitis, and has potential application value. However, CT is radiative and provides limited information. MRI has the advantages of no radiation and high resolution of soft tissue and exhibits high diagnostic value for the evaluation of complications of AP (13, 14). Moreover, there is no report on the prediction of the recurrence of AP by radiomics studies based on MRI. Therefore, the purpose of this study was to compare the diagnostic value of the radiomics feature model based on T2-weighted imaging (T2WI) MRI and the clinical feature model in predicting the recurrence of AP.

## Materials and Methods

### Materials

This study was approved by the Medical Ethics Committee of our hospital. Given the retrospective nature of the study, the ethics committee waived the need for informed consent.

**Figure 1:**
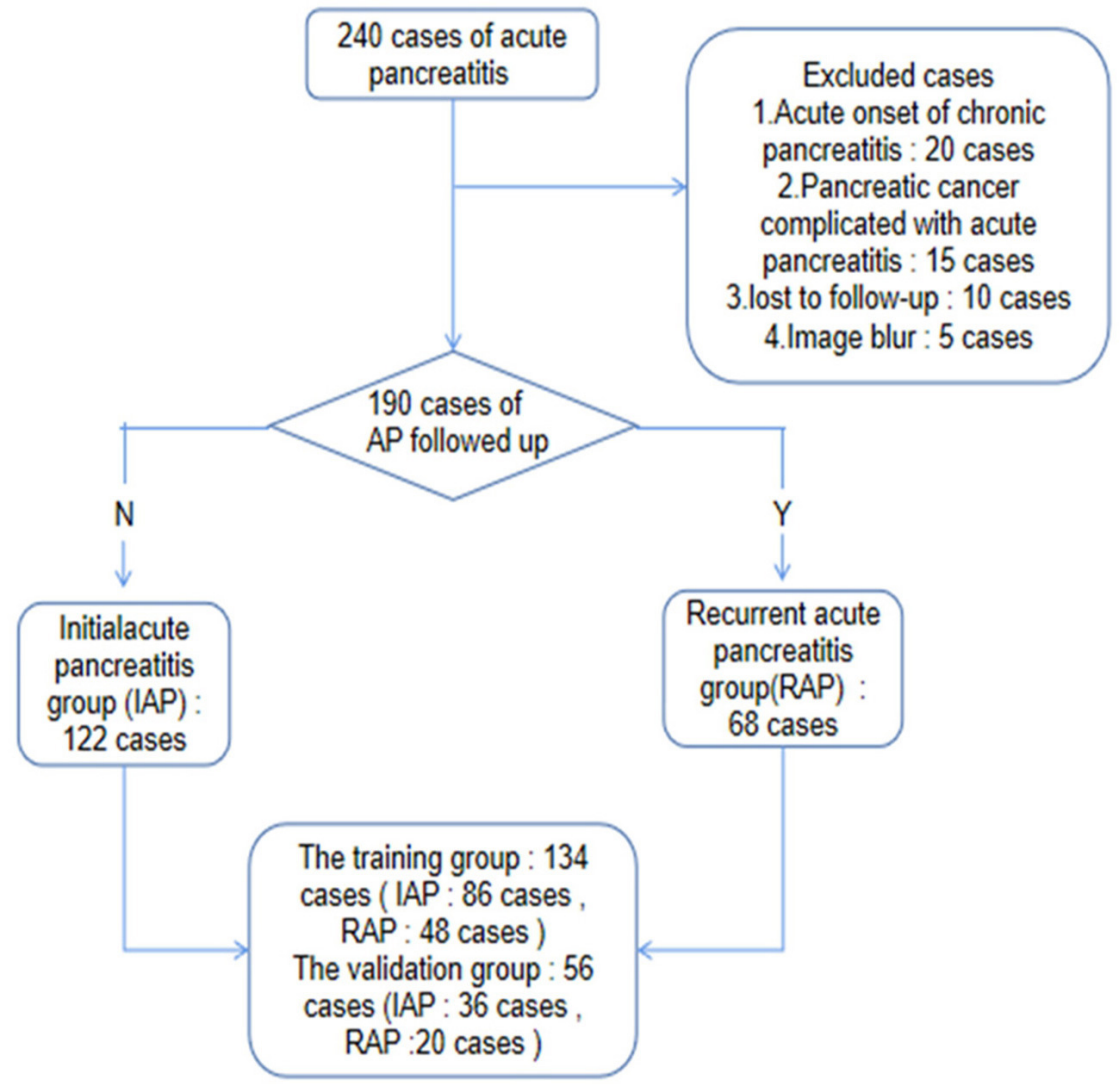
Patient selection flow chart. 240 patients with AP were collected retrospectively according to the inclusion criteria, and 190 patients were left according to the exclusion criteria. Through two-year follow-up or hospitalization records, the patients were divided into IAP group and RAP group, and then divided into training group and validation group in a ratio of 7:3.

A total of 190 patients diagnosed with AP according to the inclusion criteria in our hospital from January 2016 to December 2018 were collected retrospectively. The first episode of acute pancreatitis was determined by asking the patient's medical history. After 3 months of cure, the patients were followed up by telephone and readmission records every 3 months. The patients were divided into the IAP group and RAP group (Figure 1). Clinical features, such as sex, age, severity, complications, stones, hyperlipidemia, and drinking history, were collected.

#### Inclusion Criteria

IAP diagnostic criteria refer to the Atlanta Classification System as amended in 2012 (15). IAP can be diagnosed when two of the following three conditions are met. (1) Sudden, intense, persistent abdominal pain may be accompanied by right shoulder and lower back radiation pain. (2) Serum amylase and lipase increased abnormally to >3-fold higher than the upper limit of the normal value. (3) Typical imaging findings of AP.

The diagnostic criteria of RAP were as follows (16). (1) AP has had two or more episodes. (2) The interval between the two AP attacks is at least 3 months, during which the patients reach the standard of recovery or almost recovery.

#### Exclusion Criteria

(1) Patients with chronic pancreatitis, pancreatic tumors and other pancreatic diseases were excluded. (2) Image blur caused by respiratory motion artifact. (3) The clinical data were incomplete, and the patients were not followed up.

### Methods

#### Imaging Acquisition

All patients were examined on the day of admission by a GE Discovery MR750 3.0T MRI scanner and were imaged with 32-channel body phased array coil imaging. The parameters of axial single-shot fast spin-echo T2WI were as follows: slice thickness = 5 mm, echo time = 120 ms, repetition time = 6,000 ms, spacing = 1 mm, matrix = 320 × 256, and field of view = 34 cm × 34 cm.

#### Image Preprocessing and Analysis

##### Image Segmentation and Feature Extraction

**Figure 2:**
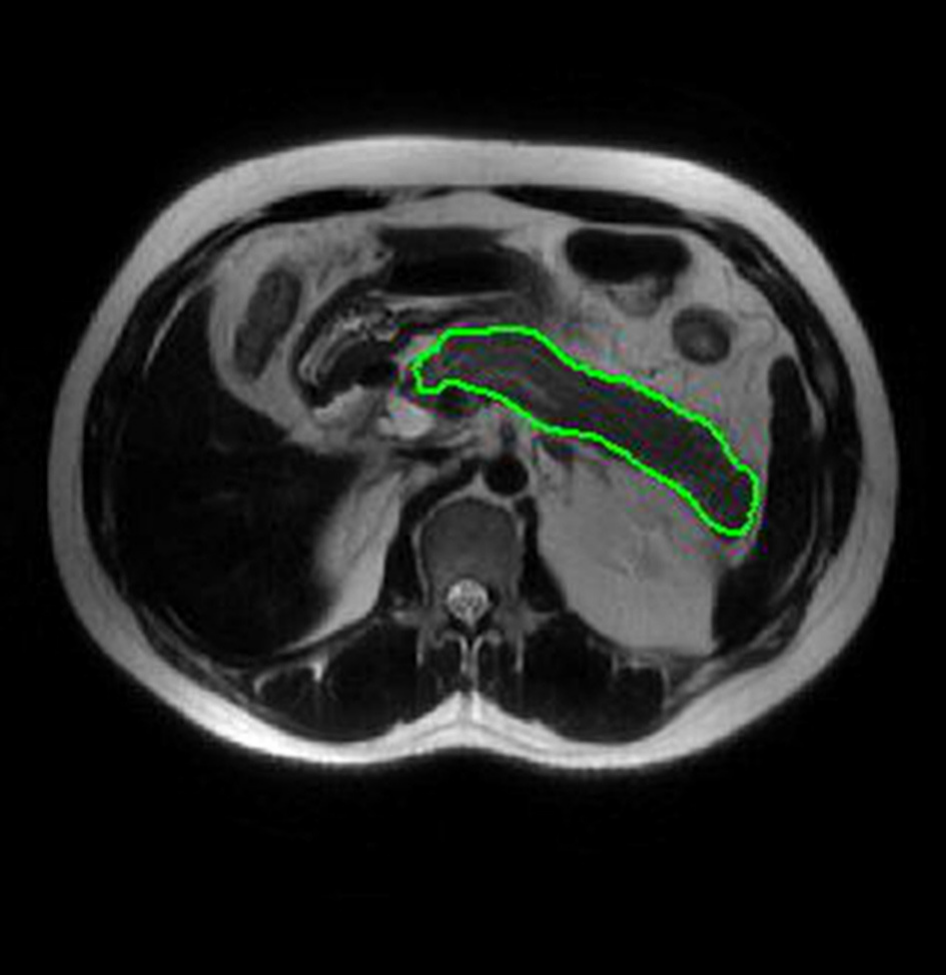
ROI placement diagram. Regions of interest (ROI) segmentation by IBEX software. Delineate three dimensional areas of interest of the pancreas, including areas of necrosis and avoiding common bile duct and blood vessels.

Two radiologists with 5 years of experience in abdominal imaging each used imaging biomarker explorer (IBEX, β1.0, http://bit.ly/IBEX_MDAnderson) software to delineate the three dimensional area of interest around the pancreatic margin including the necrotic area of the pancreas, and to avoid the common bile duct and blood vessels (Figure 2). IBEX software is an open infrastructure software platform that flexibly supports common radiomics workflow tasks and is widely used in radiomics analysis (17, 18). Five common feature groups were extracted from IBEX, including the gray cooccurrence matrix, gray length matrix, histogram and shape. A total of 1353 features were extracted from the T2WI images (Supplementary Data Sheet 1).

##### Data Preprocessing

The data in this study are derived from the same scanner and the same scanning parameters. However, the dimensions between the features are different, the center and scale are adopted to preprocess the extracted radiomics features, and box-cox transformation is also adopted to eliminate the problems of homogeneity of variance and non-normal distribution.

##### Intraobserver and Interobserver Consistency Tests

Radiologists 1 and 2 delineate the region of interest of AP and RAP initial scanned images, respectively, extracted the features and compared them to evaluate the interobserver consistency of radiomics features among the viewers. Half of the T2WI images were randomly selected from all the patients to evaluate intraobserver and interobserver consistency. After 2 weeks, the features were again outlined and extracted by radiologist 1 and compared with the first feature to evaluate the intraobserver consistency of the radiomics features. Intraclass correlation coefficients (ICCs) >0.75 were considered to indicate good consistency.

##### Dimension Reduction and Feature Screening

In this study, the least absolute contraction and selection operator (LASSO) model was optimized by 5 times 10 times cross validation method, and finally the lamda coefficient was determined to be 0.01. The filtered features are sorted, and the top five features are modeled. This process can effectively prevent the over fitting of the model and ensure the generalization of the model.

##### Model Building and Performance Evaluation

The radiomics model based on the optimal features and the combined model based on the optimum radiomics features and clinical independent risk factor were established by logistic regression model. The clinical model was established by multifactor logistic regression. The area under the receiver operating characteristic curve, sensitivity, specificity, and accuracy were used to evaluate the prediction performance of the model. Then the ability of the model is evaluated by an independent validation group.

##### Statistical Method

The data were analyzed using SPSS 23.0 software and R language (version 3.5.2, https://www.r-project.org/). Two independent samples *t*-tests or Mann-Whitney *U*-tests were used for quantitative data. The chi-squared test or Fisher's test was used for qualitative data. The Delong test was used to compare AUCs among the three models. *P* < 0.05 was considered statistically significant.

## Results

### Clinical Characteristics

Table lists the clinical characteristics of patients in the IAP group and RAP group (Table 1). A significant difference in the incidence of hyperlipidemia was noted between the IAP group and RAP group (61/122 vs. 46/68, *p* < 0.05), but there was no difference in sex, age, severity, complications, gallstones, and alcoholic etiology (all *p* > 0.05). Binary logistic regression analysis showed that hyperlipidemia was an independent risk factor for recurrence of AP, and the OR was 0.43 (95% CI: 0.220–0.849).

**Table 1:** Clinical characteristics between acute pancreatitis group and recurrent acute pancreatitis group.

	**AP (*n* = 122)**	**RAP (*n* = 68)**	***P***
**Sex**
Male	70 (57.4%)	41 (60.3%)	0.760
Female	52 (42.6%)	27 (39.7%)	
Age	46.00 (37.00–55.25)	45.50 (40.00–59.75)	0.412
**Severity**
Mild	37 (30.3%)	22 (32.4%)	0.786
Moderate	60 (49.2%)	30 (44.1%)	
Severe	25 (20.5%)	16 (23.5%)	
**Complications**
No	23 (18.9%)	26 (38.2%)	0.073
Yes	99 (81.1%)	42 (61.8%)	
**Gallstones**
No	80 (65.6%)	40 (58.8%)	0.433
Yes	42 (34.4%)	28 (41.2%)	
**Hyperlipidemia**
No	61 (50.0%)	22 (32.4%)	0.02^*^
Yes	61 (50.0%)	46 (67.6%)	
**Alcoholic etiology**
No	77 (63.1%)	46 (67.6%)	0.635
Yes	45 (36.9%)	22 (32.4%)	

* *p < 0.05*.

*AP, acute pancreatitis; RAP, recurrent acute pancreatitis*.

### ICC and Feature Screening Results

Five hundred thirteen stable features were retained through the evaluation of consistency (ICCs >0.75) between intraobservation and interobservation features.

**Figure 3:**
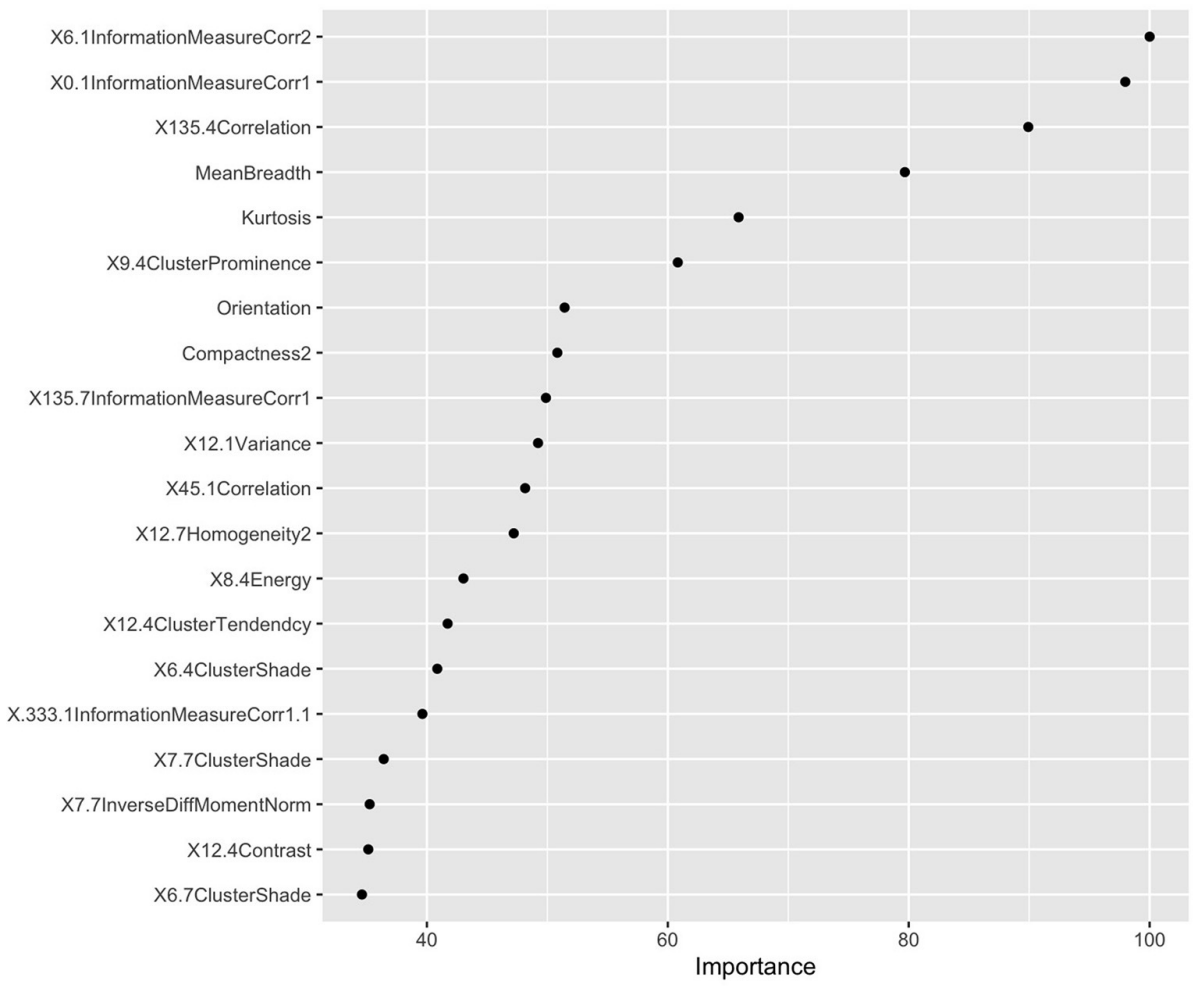
Radiomics feature screening results based on LASSO values. According to the LASSO characteristic coefficients, the features were ranked by importance.

The LASSO was used to screen five hundred thirteen features, the screening results were ranked, and the first five features were incorporated into the multi-factor logistic regression model (Figure 3).

### Establishment and Evaluation of Models

**Figure 4:**
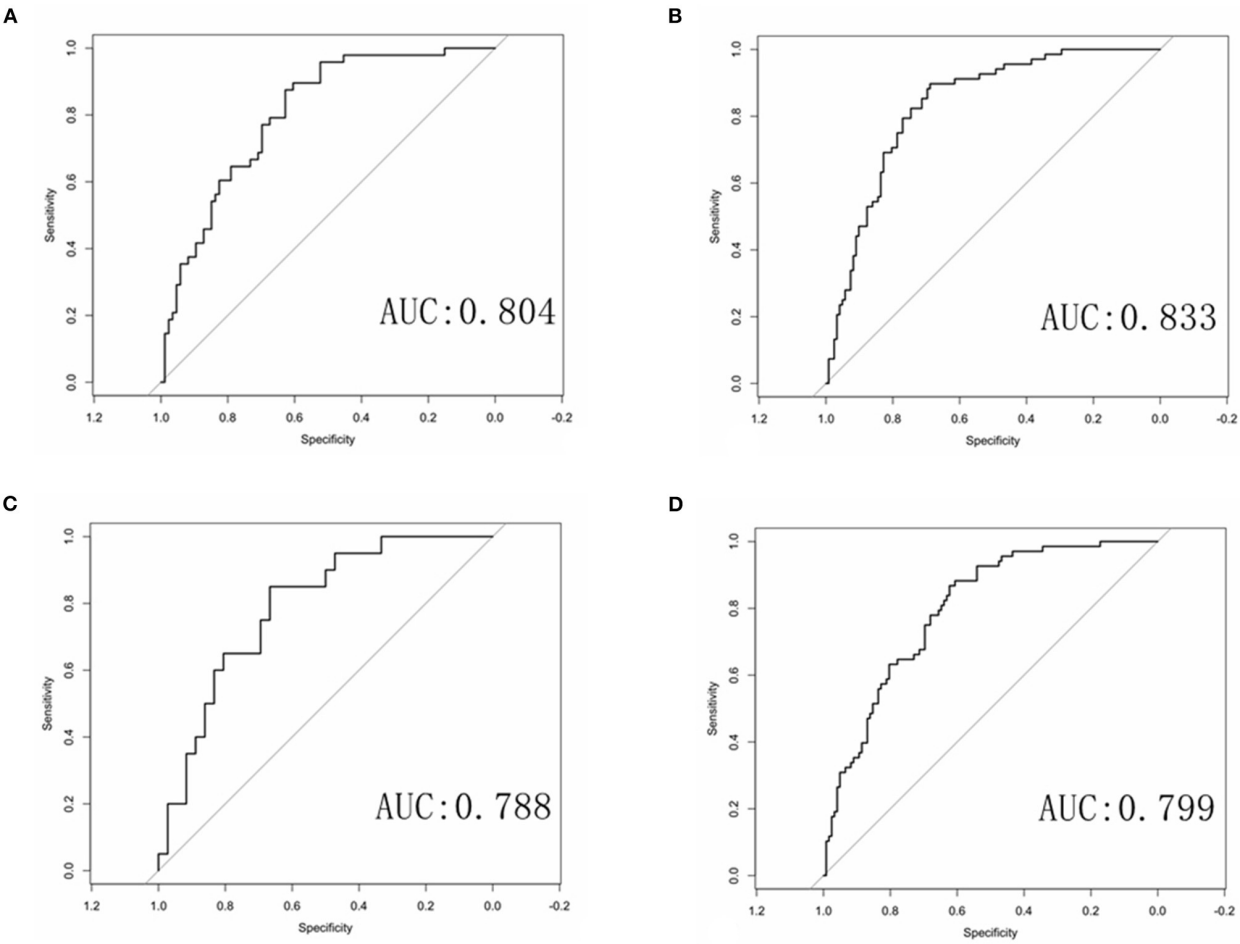
ROC curves of the radiomics and combined radiomics model. **(A)** ROC curve of radiomics model in training group. **(B)** ROC curve of combined model in training group. **(C)** ROC curve of radiomics model in validation group. **(D)** ROC curve of combined model in validation group.

**Figure 5:**
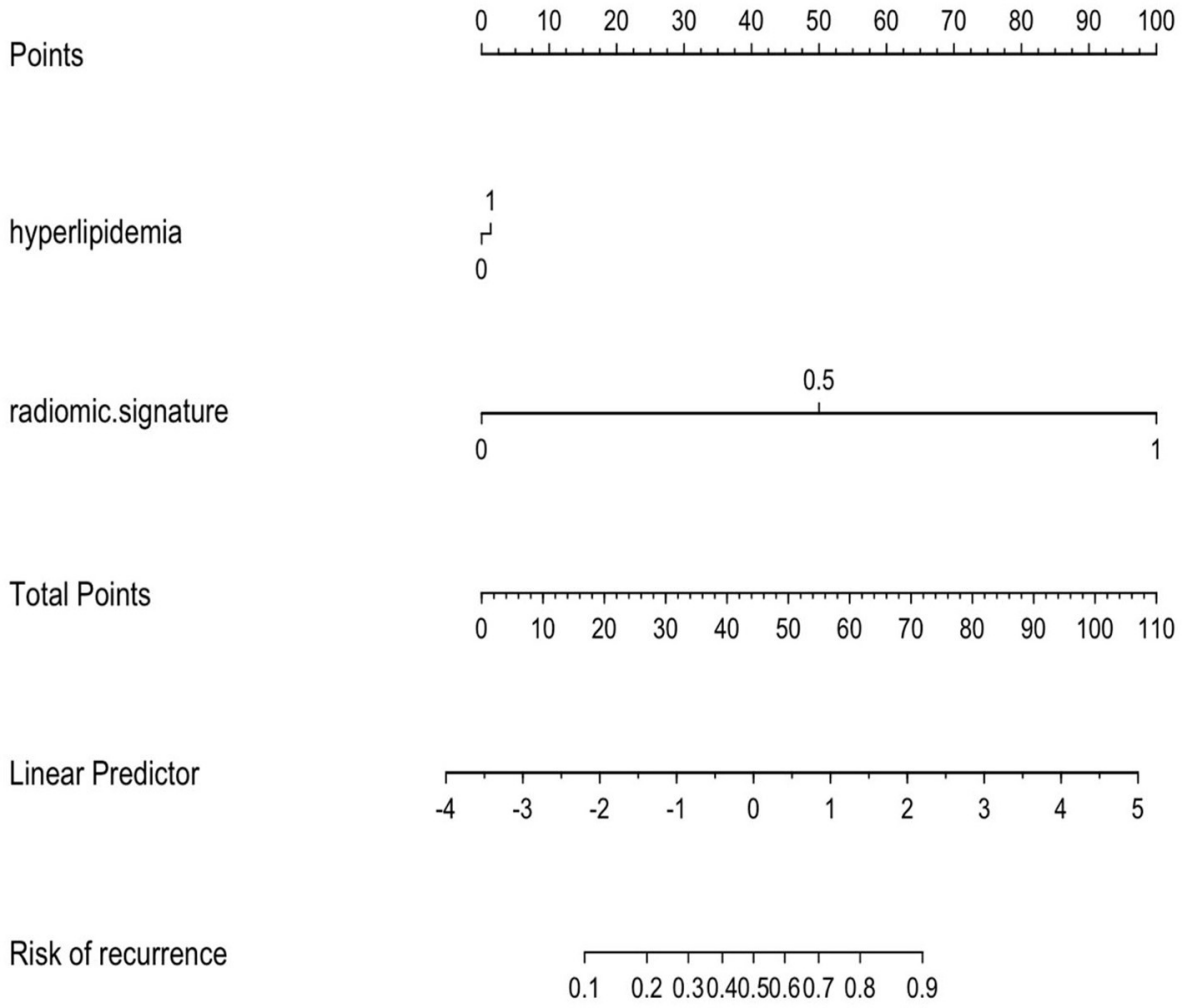
The combined model is presented as a nomogram, which incorporated hyperlipidemia and radiomics signature. Draw a vertical line on the first line to get the corresponding score. The total score of the two included features is reflected in line 5, and the last line determines the possibility of AP recurrence.

Multivariate logistic regression was used to establish radiomics model, combined model and clinical model. Finally, only four statistically significant features were included in the radiomics model. In the training group, the AUCs of the radiomics model and the combined model for predicting RAP were 0.804 and 0.833, respectively. The sensitivities of the radiomics model and combined model were 0.646 and 0.691, respectively. The specificities of the radiomics model and combined model were 0.791 and 0.828, respectively (Figures 4A,B). In the validation group, the AUCs of the radiomics model and the combined model for predicting RAP were 0.788 and 0.799, respectively. The sensitivities of the radiomics model and combined model were 0.550 and 0.618, respectively. The specificities of the radiomics model and combined model were 0.833 and 0.803, respectively (Figures 4C,D). The AUCs of clinical model in training group and validation group were 0.677 and 0.572, respectively. The sensitivities were 0.765 and 0.660, respectively. The specificities were 0.590 and 0.530, respectively (Table 2). The AUCs of the radiomics model and combined model were greater than those of the clinical model (*p* = 0.008 and *p* = 0.007, respectively.). There was no significant difference in AUC between the radiomics model and the combined model for predicting RAP (*p* = 0.067*)*. In this study, we established a nomogram of the combined model and used it to analyze the recurrence risk of patients with acute pancreatitis (Figure 5).

**Table 2:** The performance of three models for predicting recurrent acute pancreatitis in training group and validation group.

	**Training group**				**Validation group**			
	**AUC (95% CI)**	**Sensitivity**	**Specificity**	**Accuracy**	**AUC (95% CI)**	**Sensitivity**	**Specificity**	**Accuracy**
Radiomics model	0.804 (0.730–0.877)	0.646	0.791	0.739	0.788 (0.669–0.908)	0.550	0.833	0.732
Combined model	0.833 (0.776–0.891)	0.691	0.828	0.779	0.799 (0.737–0.8614)	0.618	0.803	0.737
Clinical model	0.677 (0.610–0.745)	0.765	0.590	0.653	0.572 (0.4847–0.6597)	0.660	0.530	0.534

*AP, acute pancreatitis; RAP, recurrent acute pancreatitis; AUC, area under the curve; CI, confidence interval*.

## Discussion

At present, there are no published articles that predict the recurrence of AP based on radiomics of MRI. Previous studies (19–21) mainly focused on the clinical risk factors for recurrence of AP, but few studies based on radiomics to predict recurrence of AP, and only one radiomics study (12) based on CT enhancement has been published (12). Our study not only established a clinical factor risk model, but also established a radiomics model and a combined model of radiomics features and clinical features to predict the recurrence of AP. Our results indicated that the combined model and radiomics model are superior to the clinical model in predicting the recurrence of AP. Then, the training group and validation group showed consistent high AUC both in the combined model and radiomics model, which suggested the two models are relatively stable and has a good application prospect for the prediction of recurrence of AP. In addition, we further determined the risk probability of recurrence of AP using a nomogram, which is helpful to evaluate the prognosis of AP.

In this study, 1,353 radiomics features of patients with AP were extracted from T2WI MRI sequences. Four optimal features were selected by LASSO and multivariate logistic regression, including information measure Corr 1, information measure Corr 2, mean breadth, kurtosis. Based on the four optimal features, the radiomics model and combined model are established. Our results found that the AUCs of the radiomics model and the combined model in the training group were 0.804 and 0.833, respectively. The AUCs of the radiomics model and the combined model in the validation group were 0.788 and 0.799, respectively. Both the radiomics model and the combined model showed high sensitivity and specificity. The results show that the radiomics model and the combined model are better than the clinical model in predicting the RAP. Previous study (12) confirmed this result, the radiomics model and the combined model based on enhanced CT are better than the clinical model. The reasons for the high AUC of the radiomics model and the combined model may be as follows. First, the data of patients included in this study were all from the same scanner, and the scanning sequence and parameters were highly consistent, which made the features extracted in this study more stable and less different. Second, we chose magnetic resonance imaging, which can combine multiple sequences to clearly show the anatomical relationship and inflammatory range of the pancreas, and judge the edema and bleeding of pancreatic tissue. Therefore, more features may be extracted from high-throughput data to provide more useful information for predicting the recurrence of AP (22–24).

Another interesting result of our study was that the AUC of the clinical model was inferior to that of the radiomics model and the combined model. The AUC of the clinal model in training set was 0.677. We found that the prevalence of hyperlipidemia of RAP was significantly higher than that of IAP, and hyperlipidemia was an independent risk factor for recurrence of AP. The results are consistent with previous study (12). Because there are many clinical factors affecting the recurrence of AP, it is difficult to predict the recurrence of AP exclusively based on clinical characteristics. Therefore, a clinical model combined with a radiomics model can improve the predictive value for the recurrence of AP. Moreover, based on the nomogram results, we found that compared with the clinical features, a greater proportion of the radiomics features were predictive of the recurrence risk score of AP, suggesting that the radiomics features had an advantage over independent clinical features in predicting the recurrence of AP.

There are several limitations in our study. First, the data for this study were obtained from a single center, and the scanning protocols and parameters of different centers may differ, which will lead to a poor generalization ability of the model. Second, the data of RAP in this study were obtained from a relatively small number of patients. Third, we did not compare the predictive values of radiomics models based on other MRI sequences. However, our study shows the promise of a radiomics model in predicting the recurrence of AP. Finally, the contour of the pancreas and the extent of inflammation are occasionally difficult to distinguish in AP, and it is necessary for a physician with some experience in imaging clinical practice to outline the pancreas. In addition, the boundary between the pancreas and inflammation and surrounding organs, blood vessels and bile ducts was not clear in T2WI fat suppression. Therefore, our radiomics study based on T2WI images may avoid this interference. The establishment of automatic pancreas segmentation software may help to improve this situation in the future. Therefore, it is necessary to further expand the patient data and compare the value of radiomics based on different MRI images or multiparameter MRI images in predicting the recurrence of AP.

## Conclusions

In conclusion, radiomics features based on magnetic resonance T2WI could be used as a biomarker to predict the recurrence of AP. In addition, hyperlipidemia may be an important clinical feature to predict the recurrence of AP. The combined model based on hyperlipidemia and radiomics features and the radiomics model may have important clinical value to predict the recurrence of AP as a quantitative analysis method.

## Data Availability Statement

The raw data supporting the conclusions of this article will be made available by the authors, without undue reservation.

## Ethics Statement

The studies involving human participants were reviewed and approved by Committee of the Affiliated Hospital of North Sichuan Medical College. Written informed consent for participation was not required for this study in accordance with the national legislation and the institutional requirements. Written informed consent was not obtained from the individual(s) for the publication of any potentially identifiable images or data included in this article.

## Author Contributions

YH and NL revised the manuscript for important intellectual content. KP, LT, LL, and QL collected the cases and analyzed the data. XH take final responsibility for this article. All authors contributed to the article and approved the submitted version.

## Funding

This study was funded by Sichuans Science and Technology Program (No. 2020088 to NL), the Science and Technology Project of the Health Planning Committee of Sichuan (No. 19PJ203 to NL), and Bureau of Science & Technology and Intellectual Property Nanchong City (No. 19SXHZ0429 to XH and No. 19SXHZ0255 to NL).

## Conflict of Interest

The authors declare that the research was conducted in the absence of any commercial or financial relationships that could be construed as a potential conflict of interest.

## Publisher's Note

All claims expressed in this article are solely those of the authors and do not necessarily represent those of their affiliated organizations, or those of the publisher, the editors and the reviewers. Any product that may be evaluated in this article, or claim that may be made by its manufacturer, is not guaranteed or endorsed by the publisher.

## Abbreviations

MRI: magnetic resonance imaging
T2WI: T2-weighted imaging
T1WI: T1-weighted imaging
AP: Acute pancreatitis
IAP: Initial acute pancreatitis
RAP: recurrent acute pancreatitis
AUC: Area under the receiver operating characteristic curve
ICC: Intraclass correlation coefficient
LASSO: Least absolute shrinkage and selection operator
ROI: Region of interest.
